# A new blood DNA methylation signature for Koolen-de Vries syndrome: Classification of missense *KANSL1* variants and comparison to fibroblast cells

**DOI:** 10.1038/s41431-024-01538-6

**Published:** 2024-01-29

**Authors:** Zain Awamleh, Sanaa Choufani, Wendy Wu, Dmitrijs Rots, Alexander J. M. Dingemans, Nael Nadif Kasri, Susana Boronat, Salvador Ibañez-Mico, Laura Cuesta Herraiz, Irene Ferrer, Antonio Martínez Carrascal, Luis A. Pérez-Jurado, Gemma Aznar Lain, Juan Dario Ortigoza-Escobar, Bert B. A. de Vries, David A. Koolen, Rosanna Weksberg

**Affiliations:** 1 Genetics and Genome Biology Program, Research Institute, the Hospital for Sick Children, Toronto, ON M5G 1×8 Canada; 2 Department of Human Genetics, Radboud university medical center, Donders Institute for Brain, Cognition, and Behavior, Nijmegen, The Netherlands; 3 Department of Pediatrics, Hospital del Santa Creu y Sant Pau, Barcelona, Spain; 4 Department of Pediatric Neurology, Hospital Virgen de la Arrixaca, Murcia, Madrid Spain; 5 Department of Pediatric Neurology, Hospital de Manises, Valencia, Spain; 6 Department of Genetics, Consorcio Hospital General de Valencia, Valencia, Spain; 7 Department of Pediatrics, Hospital de Requena, Valencia, Spain; 8 Genetics Unit, Universitat Pompeu Fabra, Hospital del Mar Research Institute (IMIM) and CIBERER, Barcelona, Spain; 9 Movement Disorders Unit, Institut de Recerca Sant Joan de Déu, CIBERER-ISCIII and European Reference Network for Rare Neurological Diseases (ERN-RND), Barcelona, Spain; 10 Division of Clinical and Metabolic Genetics, Department of Pediatrics, the Hospital for Sick Children, University of Toronto, Toronto, ON M5G 1×8 Canada

**Keywords:** DNA methylation, Diagnostic markers

## Abstract

Pathogenic variants in *KANSL1* and 17q21.31 microdeletions are causative of Koolen-de Vries syndrome (KdVS), a neurodevelopmental syndrome with characteristic facial dysmorphia. Our previous work has shown that syndromic conditions caused by pathogenic variants in epigenetic regulatory genes have identifiable patterns of DNA methylation (DNAm) change: DNAm signatures or episignatures. Given the role of KANSL1 in histone acetylation, we tested whether variants underlying KdVS are associated with a DNAm signature. We profiled whole-blood DNAm for 13 individuals with *KANSL1* variants, four individuals with 17q21.31 microdeletions, and 21 typically developing individuals, using Illumina’s Infinium EPIC array. In this study, we identified a robust DNAm signature of 456 significant CpG sites in 8 individuals with KdVS, a pattern independently validated in an additional 7 individuals with KdVS. We also demonstrate the diagnostic utility of the signature and classify two *KANSL1* VUS as well as four variants in individuals with atypical clinical presentation. Lastly, we investigated tissue-specific DNAm changes in fibroblast cells from individuals with KdVS. Collectively, our findings contribute to the understanding of the epigenetic landscape related to KdVS and aid in the diagnosis and classification of variants in this structurally complex genomic region.

## Introduction

Koolen-de Vries syndrome [KdVS; MIM#610443] is a rare autosomal dominant disorder characterized by developmental delay, variable degrees of intellectual disability, hypotonia, epilepsy, congenital malformations in multiple organ systems, and characteristic facial dysmorphia [1, 2]. Individuals diagnosed with KdVS have a 17q21.31 microdeletion encompassing the *KANSL1* gene or a heterozygous intragenic pathogenic variant in *KANSL1* (KAT8 regulatory NSL complex subunit 1) [1, 3–7]. Comparison of phenotypes of individuals with 17q21.31 microdeletions to that of individuals with single nucleotide *KANSL1* variants found no significant phenotypic difference [2, 3, 8]. Most of the single nucleotide variants in *KANSL1* that cause KdVS are typically de novo frameshift and nonsense variants. However, recent reports identified additional variant types that may be linked to KdVS, such as a intragenic *KANSL1* microduplication and a missense *KANSL1* variant. In addition to the diagnostic challenges of classifying missense *KANSL1* variants, the structural complexity and genomic variation of the 17q21.31 region present additional diagnostic challenges [9]. The complexity of mapping and interpretation of both copy number and single nucleotide variants is a result of low copy repeats or segmental duplications flanking the 17q21.31 region resulting in multiple haplotypes [10].

The *KANSL1* gene encodes an intrinsically disordered protein that functions as a part of the nonspecific lethal (NSL) complex involved in epigenetic and global transcription regulation [11, 12]. The NSL complex is an evolutionarily conserved, chromatin modifying, multiprotein assembly responsible for the acetylation of lysine 16 on histone 4 (H4K16ac) [11, 13]. The acetylation reaction utilizes acetyl-CoA and results in transcriptionally active chromatin [11]. The NSL complex is widely expressed within cells and acts broadly to regulate organismal development and cellular homeostasis [11, 14, 15]. The unstructured KANSL1 protein is highly expressed in many tissues and acts as a scaffolding protein for members of the NSL complex with predicted binding regions for KAT8 and WDR5 [14].

Our research group and others have reported unique changes in DNA methylation (DNAm) in genes encoding epigenetic regulators, termed ‘DNAm signatures’ or ‘episignatures’ [16–22]. DNAm signatures are gene- and disorder-specific, and to date, DNAm signatures for more than 50 neurodevelopmental syndromes have been defined, most of which are caused by pathogenic variants in genes encoding epigenetic regulators [23, 24]. These DNAm signatures are likely established via crosstalk between histone modifications and DNAm. Although the exact molecular mechanisms underpinning DNAm signatures are not yet fully elucidated, a rapidly expanding body of work has emerged demonstrating the diagnostic utility of DNAm signatures in the classification of variants of uncertain significance (VUS). For VUS classification, the DNAm profile for a single case is compared to a gene specific DNAm signature, derived from analysis of samples for patients with pathogenic variants within the gene in question [16]. We generated and validated a unique DNAm signature for KdVS from 12 individuals with pathogenic *KANSL1* variants and 4 individuals with 17q21.31 microdeletions. We demonstrate the diagnostic utility of the signature and classify two *KANSL1* VUS as well as four variants in individuals with atypical clinical presentation. Lastly, we identify tissue-specific methylation changes in fibroblast cells from individuals with KdVS.

## Methods

### Research Participants

Informed consent was obtained from all research participants and/or their guardian(s) according to the protocol approved by the Research Ethics Board of the Hospital for Sick Children (REB#1000038847). Individuals were recruited through our International Epigenetic Consortium (IEC), which includes both local and international collaborators. Following recruitment, the cohort consisted of 17 individuals with KANSL1 variants, 5 individuals with 17q21.31 CNVs (total *n* = 22). The patient demographics, variant and clinical information are presented in Supplementary Table S1. Of the 22 individuals in the study 16 individuals had clinical features in line with KdVS and pathogenic *KANSL1* variants or 17q21.31 microdeletions encompassing *KANSL1*, the remaining 6 individuals had VUS and/or atypical phenotypes. Banked DNA samples from age- and sex-matched typically developing individual (*n* = 200) were included as control subjects (Supplementary Table S2). These individuals were recruited from the Hospital for Sick Children and the Province of Ontario Neurodevelopmental Disorders (POND) Network and were deemed typically developing (Dr Gregory Hanna). ‘Typically developing’ was defined as healthy and developmentally normal by using formal cognitive/behavioral assessments (POND) or via physician/parental screening questionnaires (SickKids).

### DNAm profiling and data processing

Genomic DNA was extracted from peripheral blood and bisulfite converted using the EpiTect PLUS Bisulfite Kit (QIAGEN, Valencia, CA) according to the manufacturer’s protocol. Sodium bisulfite converted DNA was then hybridized to the Illumina Infinium Human Methylation EPIC BeadChip at The Center for Applied Genomics (TCAG), Hospital for Sick Children Research Institute, Toronto, Canada. The minfi Bioconductor package in R was used to preprocess data including quality control, Illumina normalization and background subtraction, followed by extraction of beta (β) values as previously described [25]. Standard quality control metric in minfi were used, including median intensity QC plots, density plots and control probe plots. Probes with detection flaws (*n* = 998), probes near SNP with minor allele frequencies above 1% (*n* = 177660), cross-reactive probes (*n* = 30749), probes with raw beta of 0 or 1 in > 25% of samples (*n* = 239), non-CpG probes (*n* = 2497) [26], and X and Y chromosome probes (*n* = 19627) were removed, *n* = 639490 probes remained for differential methylation analysis.

### DNAm signature generation

We identified differentially methylated CpG sites in whole blood derived DNA from *n* = 8 individuals with truncating variants in *KANSL1* or pathogenic 17q21.31 microdeletions and a clinical diagnosis of KdVS compared to 21 sex- and age-matched typically developing controls (Supplementary Tables S1 and S2). We applied the blood cell-type proportion estimation tool in minfi (Supplementary Fig. 1). We identified differentially methylated CpG sites using Limma [27] regression modeling with age, sex, and cell type proportions [28] as covariates as previously described [21]. The thresholds used for the identification of significant CpG sites were Benjamini–Hochberg adjusted *p*-value < 0.05 and a |Δβ|> 10%. Principal component analysis (PCA) and hierarchical clustering were generated using Qlucore Omics Explorer V3.8 (QOE, www.qlucore.com).

### Gene ontology

The list of CpG positions comprising the DNAm signature was submitted to GREAT [29] for Gene Ontology (GO) enrichment analysis. Enrichment of each GO term was calculated using a foreground/background hypergeometric test over genomic regions, using the set of CpG sites after minfi probe quality control as a background set (*n* = 639490). Overlapping genes were mapped using default GREAT settings with the following exceptions: the cut-off to annotate a CpG as overlapping with a gene (‘distal gene mapping’ setting) was set to 10 kb, and only terms enriched with three of more gene hits and FDR < 0.05 were reported.

### Machine learning classification models

We developed a classifier using a support vector machine (SVM) learning models trained on the KdVS DNAm signature using the R package ‘caret’. The SVM model, was set to ‘probability’ mode to generate probability scores ranging between 0 and 1 (0–100%), classifying variants as ‘KdVS-like’ (score > 0.5) or ‘control-like’ (score <0.5).

### In silico predictions and protein modeling

All gene variant annotations were generated with Alamut visual 2.11 and the schematic of the *KANSL1* gene structure and variant location was generated using ProteinPaint. Multiple tools were used for in silico predictions for missense VUS in *KANSL1* including PolyPhen-2 [30], SIFT [31], MutationTaster [32], and CADD [33]. To further understand the effects of *KANSL1* missense variants on the KANSL1 protein we utilized 3D protein modeling. We modeled a 3D KAT8-KANSL1 protein complex using ab initio modeling in AlphaFold2-multimer as implemented in ColabFold. For the initial complex structure prediction, we used the full KAT8 protein sequence and the C-terminal portion of KANSL1 (amino acids 850–1105) predicted to interact with KAT8. In the final structure the C-terminal portion was further reduced to only include residues with sufficient quality (AlphaFold2 pLDDT score > 50), and non-interacting amino acid residues were removed from the final structure. To verify the predicted KAT8-KANSL1 structure, we compared it to an orthologous protein complex with solved crystal structures, the MOF-MSL1 complex (PDB: 4DNC and 2Y0M). The AlphaFold2-multimer predicted KAT8-KANSL1 complex structure was similar to the two MOF-MSL1 complex structures (RMSD = 1.02 and 0.92, respectively). The structural analyses and visualisation were performed using YASARA-structure software.

### Quantitative phenotypic analysis using PhenoScore

We utilized PhenoScore [34] to quantify phenotypic similarity between individuals EX17-EX22 and known individuals with KdVS. With PhenoScore [34], a machine learning model is trained on phenotypic data of individuals of a genetic syndrome and control individuals, to quantify phenotypic similarity using artificial intelligence. Novel, previously unseen, individuals can then be classified using the trained model to determine whether they are more similar to the syndrome or to the control group. For a comprehensive description of the methodology of PhenoScore [34], see citation.

### DNAm profiling of fibroblast cells

Fibroblast DNAm profiles were generated for individuals with truncating *KANSL1* variants and a clinical diagnosis of KdVS (*n* = 3) and controls (*n* = 7). The DNA samples were processed through the Illumina EPIC array pipeline in the same manner as blood DNA samples (see above). We identified differentially methylated CpG sites using Limma regression modeling. The thresholds used for the identification of significant CpG sites were Benjamini–Hochberg adjusted *p* value < 0.05 and a |Δβ|> 10%. Δβ represents the difference in mean DNAm (β) between groups. As part of a sub-analysis, to reduce the burden of multiple hypothesis testing, we filtered probes to include most variable probes (>10%) across all samples (295415 probes remained).

## Results

### Study cohort

**Fig. 1:**
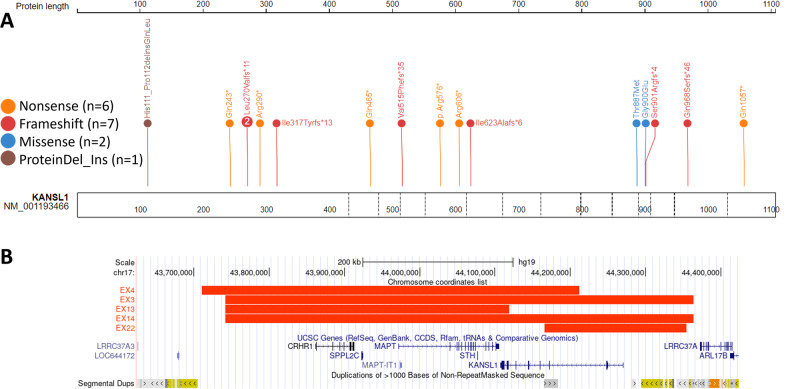
Schematic representation of genotype information for all individuals included in this study. **A** Schematic of the genomic location and type of single nucleotide variant in KANSL1 [Hg19, NM_001193466.4] generated using ProteinPaint. The number in each lollipop represents the number of individuals with that variant. **B** Schematic of the size of microdeletions in four individuals in this study generated using UCSC Genome Browser. Individuals EX1-EX8 are a part of the discovery cohort and individuals EX9-EX16 are part of the validation cohort.

We report 12 individuals with pathogenic variants in *KANSL1* (NM_001193466.4) and four individuals with typical 17q21.31 microdeletions and a clinical diagnosis of KdVS (total *n* = 16) (Table 1 and Supplementary Table S1). Pathogenic variants are predicted to adversely impact protein function and are classified as pathogenic using the ACMG variant classification guidelines. They include four nonsense variants, seven frameshift variants, and one multi-exon duplication. The reported sizes of the microdeletions at 17q21.31 causing full or partial deletion of *KANSL1* varied in our cohort between 378 Kb and 622 Kb. We report on two individuals with missense VUS in *KANSL1* (EX17–18), as well as an additional four individuals with atypical genotype-phenotype correlations involving *KANSL1* or the 17q21.31 region, and these include one individual with a mosaic nonsense *KANSL1* variant (EX19), one individual with a C-terminus nonsense variant (EX20), one individual with a deletion-insertion variant in the second exon of *KANSL1* (EX21), and one individual with an inherited 189 Kb 17q21.31 microduplication (EX22). The latter six samples (EX17-EX22) were considered for classification using the newly generated DNAm signature. Figure 1 provides a schematic representation of the *KANSL1* gene structure and variant location.

**Table 1:** Demographic and mutation information for individuals with *KANSL1* [Hg19, NM_001193466.4] or 17q21.31 [Hg19] variants included in this study.

Subject_ID	Sex	Age (years)	aachange	Category
EX1	M	8	p.(Arg606ter)	Discovery
EX2	M	3	p.(Val515Phefster35)	Discovery
EX3	M	11	17q21.31 microdeletion	Discovery
EX4	F	15.5	17q21.31 microdeletion	Discovery
EX5	F	5	p.(Ile623Alafster6)	Discovery
EX6	F	10	p.(Arg576ter)	Discovery
EX7	M	4.5	p.(Ser901Argfster4)	Discovery
EX8	F	10	p.(Gln465ter)	Discovery
EX9	F	4	p.(Ile317Tyrfster13)	Validation
EX10	F	45	p.(Leu270Valfster11)	Validation
EX11	M	16	p.(Leu270Valfster11)	Validation
EX12	F	12	p.(Gln968Serfster46)	Validation
EX13	F	5.5	17q21.31 microdeletion	Validation
EX14	M	2.7	17q21.31 microdeletion	Validation
EX15	F	3	Exon 11–13 duplication	Validation
EX16	F	3.6	p.(Arg290ter)	Validation
EX17	M	11	p.(Gly900Glu)	Classification (missense)
EX18	M	3	p.(Thr887Met)	Classification (missense)
EX19	F	23	p.(Gln243ter)	Classification (atypical)
EX20	M	0.33	p.(Gln1057ter)	Classification (atypical)
EX21	M	7	p.(His111_Pro112delinsGlnLeu)	Classification (atypical)
EX22	F	9	17q21.31 microduplication	Classification (atypical)

### KdVS DNAm signature generation

**Fig. 2:**
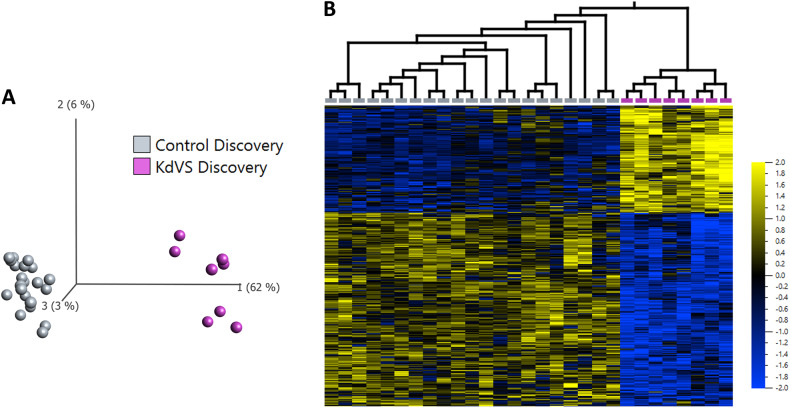
Haploinsufficiency in *KANSL1* causing KdVS is associated with a distinct DNAm signature. **A** Principal component analysis (PCA) and **B** heatmap showing clustering of the KdVS discovery subjects (*n* = 8; purple) and control discovery subjects (*n* = 21; grey) using DNAm values at the 456 CpG sites identified in the KdVS DNAm signature. The heatmap color gradient indicates the normalized DNAm value ranging from −2.0 (blue) to 2.0 (yellow). Euclidean distance metric is used in the heatmap clustering dendrograms.

We profiled genome wide DNAm in 8 individuals with a clinical diagnosis of KdVS (EX1–8). The discovery cohort included two individuals with 17q21.31 microdeletions and six individuals with pathogenic *KANSL1* variants [Hg19, NM_001193466.4] (Table 1 and Supplementary Table S1). There were no significant DNAm differences between individuals with 17q21.31 microdeletions and individuals with pathogenic *KANSL1* variants (adjusted *p*-value = 0.34). The KdVS discovery cohort (*n* = 8) included four females and four males with mean age at sample collection of 8.4 ± 4.1 years (range 3–15 years). The 21 sex- and age-matched control individuals included 10 females and 11 males with a mean age at sample collection of 8.5 ± 4.4 years (range 2–15 years) (Supplementary Table S1). We identified 456 differentially methylated CpG sites that met a threshold of false discovery rate (FDR) < 0.05 and |β|>0.10 (10% DNAm difference, Supplementary Table S3). We visualized DNAm data using principal component analysis and hierarchal clustering (Fig. 2). Approximately 62% of CpG signature sites overlapped CpG islands or shores (within 2 kb of islands). This was significantly higher than the percentage of total probes on the array mapping to islands and shores (37%) (*P* value = 4.9E−3; hypergeometric test).

### Independent validation of KdVS DNAm signature

**Fig. 3:**
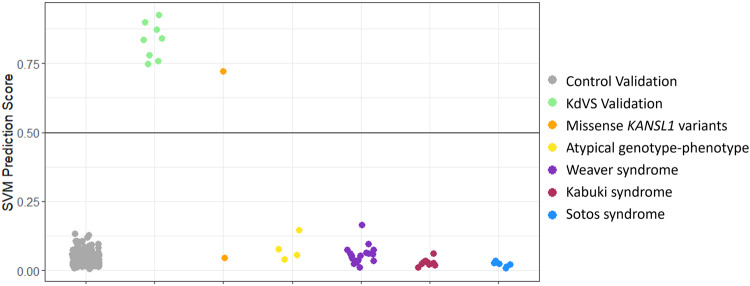
Classification of variants using SVM machine learning model based on the KdVS DNAm signature. Sample groups were scored using the KdVS support vector machine (SVM) model. KdVS syndrome validation subject (*n* = 8) classified as KDVS-like demonstrating high sensitivity of the model. Whereas validation control subjects (*n* = 200) classified with low probabilities demonstrating high specificity of the model. One KANSL1 missense variant [p.(Gly900Glu); EX17] has a control-like probability score and the other missense variant [p.(Thr887Met); EX18] has a KdVS-like probability score. Four additional variants in KANSL1 and the 17q21.31 region in individuals [EX19-22] with atypical genotype-phenotype correlations all have control-like probability scores.

We trained a machine learning classification model to robustly categorize variants as ‘KdVS-like’ or ‘control-like’ based on DNAm levels at signature sites. The model generated a probability ranging from 0 ‘control-like’ to 1 ‘KdVS-like’ for each sample (Supplementary Table S4). We classified a validation cohort of eight individuals with pathogenic sequence variants (*n* = 6) and 17q21.31 microdeletions (*n* = 2) encompassing *KANSL1* (Table 1). The SVM model generated high pathogenicity scores (75–92%) for all samples in the validation cohort demonstrating high sensitivity of the signature (Fig. 3 and Supplementary Table S4). Clustering pattern of DNAm profiles at the KdVS signature using principal component analysis and hierarchal is concordant with scoring generated from the machine learning model (Supplementary Fig. 2). We included DNAm data for an additional 200 controls (41% females, and ages 1–42 years), all of which had low SVM scores (0–13%) demonstrating high specificity of the signature (Fig. 3). We also classified three cohorts of individuals with Sotos, Weaver, and Kabuki syndromes, caused by variants in genes encoding the epigenetic regulators, *NSD1*, *EZH2*, and *KMT2D*, respectively. All three cohorts had pathogenicity scores within the control range (1–9%).

### Ontology of DNAm signature sites

Gene ontology analyses can be used to describe the role of gene targets in two biological domains: larger processes accomplished by multiple molecular functions (biological processes) and molecular-level activity of gene products (molecular functions). There were 222 unique genes that overlapped the signature sites, with 19 genes overlapping three or more CpG sites. Three genes were associated with six or more significant CpG sites and they are: *WT1* (24 sites, Supplementary Fig. 3), *LRRC27* (6 sites), and *SHANK2* (6 sites). We identified significant enrichment (FDR < 0.05, gene hits ≥ 3) for 157 biological processes and 11 molecular functions (Supplementary Tables S5 and S6).

### Classification of VUS

**Fig. 4:**
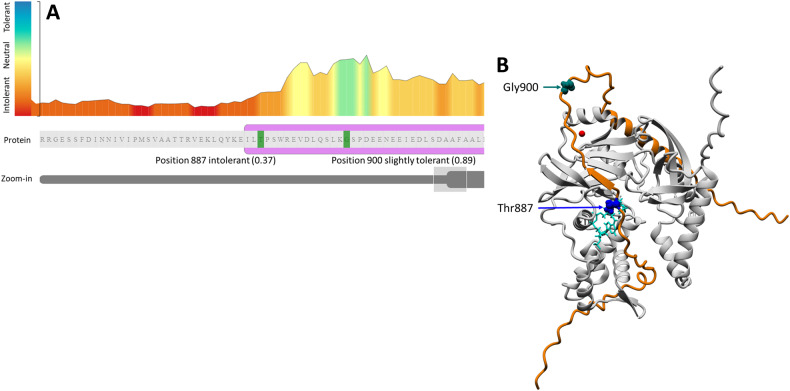
KANSL1 protein tolerance landscape and predicted model structure. **A** The diagram illustrates the landscape of KANSL1’s tolerance to missense changes according to MetaDome web server. Positions for missense variants analyzed in the present work are indicated in green. **B** The predicted structure which includes KAT8 shown in grey, acetylCoA in cyan, Zn in red, and the KANSL1 fragment in orange with Thr887 residue is show in dark blue, and Gly900 in dark green.

We classified two individuals with missense variants, individuals EX17 and EX18. The machine learning model generated a pathogenicity score within control range for individual EX17 [p.(Gly900Glu), 4.7%]. All *in-silico* predictions indicate that the variant p.(Gly900Glu) is benign and has a CADD score of 19.6 (Table 2). We also used the MetaDome web server to assess KANSL1’s protein tolerance in genomic regions overlapping the VUS reported here (Fig. 4A); the lower the score, the more intolerant the protein is to variation. Variation at amino acid position 900 was found to be ‘slightly tolerant’ with a score of 0.89.

**Table 2:** Predicted pathogenicity of missense *KANSL1* [Hg19, NM_001193466.4] variants using in silico tools generated with Alamut.

ID	Gene	Aa_change	type	SIFT	Polyphen	MutationTaster	CADD	REVEL Score	SVM %
EX17	*KANSL1*	p.(Gly900Glu)	missense	Deleterious	Benign	Benign	19.6	0.16	4.1%
EX18	*KANSL1*	p.(Thr887Met)	missense	Tolerated	Probably Damaging	Deleterious	26.7	0.74	70%

For individual EX18, the variant is de novo and the model generated a pathogenicity score within KdVS range [p.(Thr887Met), 72%]. The *in-silico* predictions for the variant p.(Thr887Met) indicate it is likely deleterious with a CADD score of 26.7 (Table 2). In MetaDome the variation at amino acid position 887 was found to be ‘intolerant’ with a score of 0.37.

Since the KANSL1 protein is intrinsically disordered, we utilized ab initio modeling using AlphaFold2-Multimer to generate a predicted KAT8-KANSL1 complex with high confidence. Based on the predicted structure, KANSL1 has multiple contiguous sites for KAT8 interaction, one of which spans positions p.860–940 overlapping the two missense VUS reported in this study. The predicted 3D protein complex model shows the Thr887 codon is located on one of the interaction surfaces with KAT8 (in grey), whereas the Gly900 codon is located on the flexible loop between two stretches of KANSL1 interacting with KAT8 (Fig. 4B).

From a clinical perspective, individual EX17 [p.(Gly900Glu), 4.7%] has developmental delay, intellectual disability, short stature, microcephaly, and a congenital malformation of the aortic arch. He has upslanted palpebral fissures, but otherwise no facial characteristic of KdVS. Individual EX18 [p.(Thr887Met), 72%] has mild developmental delay and a history of infantile spasms early in life. He had mild language delay at first but caught up. He has facial hypotonia, a long face, and full nasal tip. We used PhenoScore [34] for quantitative phenotypic analysis which combines facial photographs and phenotypic data in Human Phenotype Ontology terms (Table S7). The combined PhenoScore [34] for individual EX17 was control-like (0.13; control-like), with both face- and other phenotypic data not matching with the KdVS phenotype. The PhenoScore [34] based on facial photographs for individual EX18 was considered a phenotypic match (0.71), whereas the Phenoscore based on HPO terms was control-like (0.15).

### Classification of individuals with atypical phenotypic presentation

We classified four additional individuals with unresolved genotype-phenotype correlations. Individual EX19 clinically presented with intellectual disability, epilepsy and sleep problems but no other KdVS specific phenotypic features and had a low combined PhenoScore [34] (0.04), corresponding with a control classification. Genetic testing identified individual EX19 had a de novo mosaic variant in exon 1 of *KANSL1* [p.(Gln243ter)] as well as a frameshift variant in *SYNGAP1*. The SVM model generated a pathogenicity score within the control range for individual EX19 (3.9%). Individual EX20 was born prematurely with unilateral cleft palate and later presented with developmental delay, hearing loss, autism spectrum disorder, and recurrent infections. Genetic testing identified individual EX20 had a de novo variant in the last exon of *KANSL1* [p.(Gln1057ter)] as well as a pathogenic maternally inherited missense variant in *AMOTL1* [35]. The pathogenicity score generated by the SVM model for individual EX20 was control-like (14.6%). The combined PhenoScore [34] for individual EX20 was intermediate (0.43) due to the overlap in HPO terms (0.69) and not facial dysmorphology (0.06). Individual EX21 was born large for gestational age and later presented with mild intellectual disability, delayed speech, hydronephrosis, and facial dysmorphia noted to be inconsistent with KdVS. Genetic testing identified individual EX21 has a deletion-insertion variant in the second exon of *KANSL1* [p.(His111_Pro112delinsGlnLeu)]. The pathogenicity score generated by the SVM model for individual EX21 was within the control range (5.7%), in-line with a low combined PhenoScore [34] (0.14).

Lastly, individual EX22 presented clinically with intellectual disability, autism spectrum disorder, hypotonia, delayed psychomotor development, epilepsy, spina bifida occulta, hyperphagia and her dysmorphic features were noted to be inconsistent with KdVS. Genetic testing using aCGH for this patient, her clinically affected brother, and unaffected parents identified they each have copy number variants (CNV) within 17q21.31 overlapping the first 2 exons of *KANSL1*. The mother and father have segmental duplications (x3), whereas the brother has a segmental deletion. Individual EX22, the daughter, has a segmental duplication that encompasses the affected regions found in her family members. Samples from the mother, father and brother were not available for DNAm testing. The pathogenicity score generated by the SVM model for individual EX22 was within the control range (7.6%), again corresponding with a low, control-like, combined PhenoScore [34] (0.08).

### DNAm of fibroblast cells

**Fig. 5:**
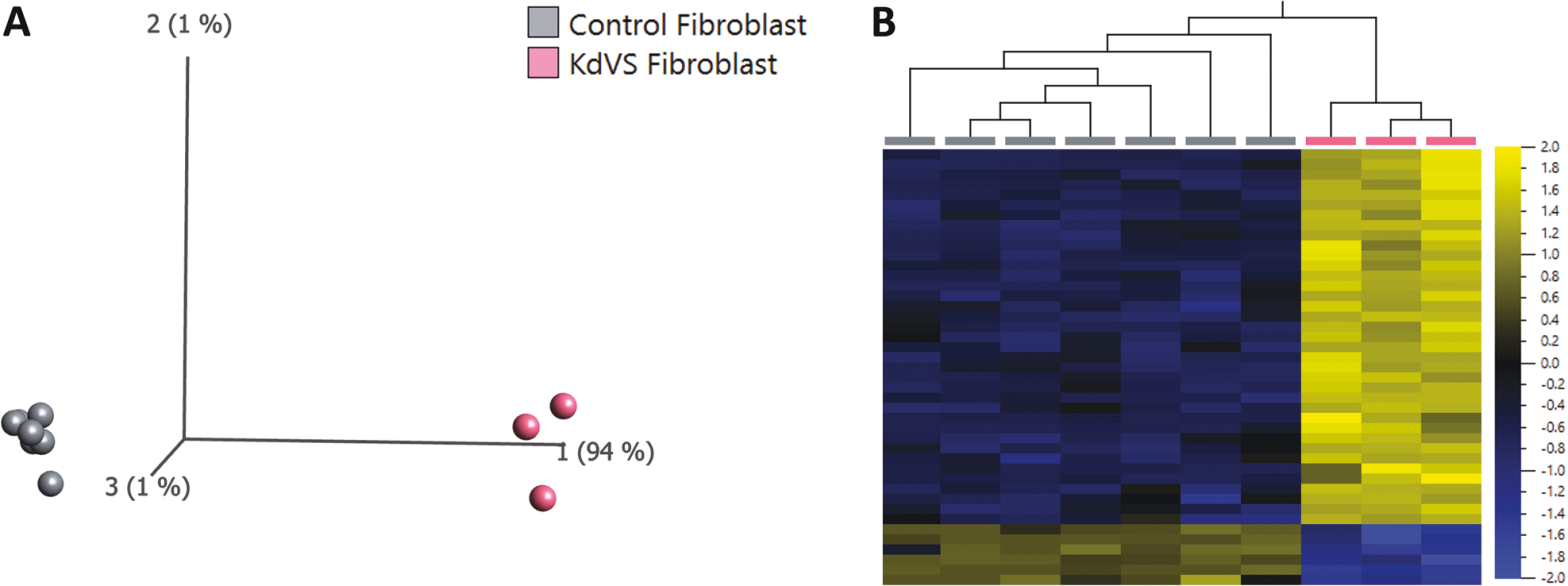
Significant DNA methylation changes in fibroblast cells from individuals with KdVS. **A** Principal component analysis (PCA) and **B** heatmap showing clustering of the KdVS discovery subjects (*n* = 3; pink) and control discovery subjects (*n* = 7; grey) using DNAm values at the 43 significant CpG sites identified. The heatmap color gradient indicates the normalized DNAm value ranging from −2.0 (blue) to 2.0 (yellow). Euclidean distance metric is used in the heatmap clustering dendrograms.

To assess tissue-specific differences we assayed DNAm in fibroblast cells from three individuals with pathogenic variants in *KANSL1* and seven typically developing ‘control’ individuals (Supplementary Table S8). At the blood derived 456 signature CpG sites, the DNAm profiles of fibroblasts from individuals with *KANSL1* variants were modestly separated from the DNAm profiles of fibroblasts from typically developing controls with the first principal component at 23% (Supplementary Fig. 4A). We conducted differential methylation analysis in fibroblasts and identified 43 significant CpG sites at a FDR < 0.05 and |β|>0.10 (Fig. 5 and Supplementary Table S9). We compared the blood- and fibroblast derived DNAm sites and identified one common CpG site as well as three common underlying genes (*APC2, ORMDL3, REEP3*). We reduced the burden of multiple hypothesis testing and filtered probes to only include those most variable across all samples (Supplementary Fig. 4B). We identified a larger number of significant CpG sites (1234) at a FDR < 0.05 and |β|>0.10 that can clearly separate KdVS fibroblast cells from control fibroblast cells by 85% on the first principal component. We also identified an additional five CpG sites overlapping the blood signature sites (Supplementary Fig. 4C).

## Discussion

In this study, we identified a unique DNAm signature associated with truncating pathogenic variants in *KANSL1* and 17q21.31 microdeletions encompassing *KANSL1* in peripheral blood of individuals clinically diagnosed with KdVS. Our findings contribute to the understanding of the epigenetic landscape related to KdVS and aid in the diagnosis and classification of variants in this complex genomic region. In this study we generated a KdVS specific DNAm signature with an expanded signature discovery cohort (*n* = 8) that included six individuals with single truncating *KANSL1* variants and two individuals with 17q21.31 microdeletions. We validated the 456 CpG site signature in an independent cohort (*n* = 8) of individuals with KdVS, which included an additional six individuals with single truncating variants and two individuals with 17q21.31 microdeletions. A preliminary blood KdVS DNAm signature based on six individuals with pathogenic *KANSL1* variants has been previously published [36]. We identified 30 CpG sites that overlap between the previously published signature and the signature generated here. We interpret this minimal overlap to demonstrate that no single signature is inclusive of all methylation changes in these patients; this is consistent with previous comparisons of DNAm signatures [37]. In part, DNAm signatures are a product of the cohort used to derive them as well as the specific methods used to select a set of differentially methylated CpGs. This is why it is so crucial to always include an independent validation cohort to test predictive efficacy, and especially when signatures are being developed for diagnostic purposes.

The machine learning model generated pathogenicity scores >75% for the KdVS validation cohort, and pathogenicity scores <13% for the control cohort, demonstrating the signature’s high sensitivity and specificity. When we compared DNAm profiles of individuals with single nucleotide variants to DNAm profiles of individuals with 17q21.31 microdeletions, there were no significant methylation differences, indicating that single variants in *KANSL1* likely cause haploinsufficiency via nonsense-mediated decay (NMD). We previously observed the same phenomenon in our DNAm studies of Kleefstra syndrome and KBG syndrome [22, 38]. These data support the hypothesis that the DNAm profiles observed in individuals with CNVs are likely driven by haploinsufficiency of a gene mapping within the CNV and has a functional role in epigenetic regulation.

Using a machine learning model based on the KdVS signature, we classified the DNAm profiles of two individuals with *KANSL1* VUS. Individual EX17 [p.(Gly900Glu)] clinically presented with developmental delay and intellectual disability but no KdVS-specific facial dysmorphia and had a control-like DNAm classification score [4.7%]. In agreement with findings from *in-silico* prediction tools [CADD score < 20], a low PhenoScore of 0.13, and protein modeling which predict variants at p. Gly900 would not significantly alter KANSL1’s structure when interacting with KAT8. Individual EX18 [p.(Thr887Met)] is mildly affected with some facial dysmorphia in-line with KdVS. The PhenoScore [34] based on facial photographs of individual EX18 identified them as a phenotypic match for KdVS (0.71). This phenotypic finding was supported by a KdVS-like DNAm classification score for individual EX18 (72%), and was in agreement with predictions from *in-sili*co tools [CADD score > 25] and protein modeling which showed the p. Thr887 codon is directly within the interacting surface near acetyl-CoA, a molecule required for the acetylation reaction. Therefore, mutations at this amino acid position [p. Thr887] are likely to impact protein-protein interactions. For both individuals EX17 and EX18, the combination of phenotype, genotype and epigenotype data was vital to determine whether the variant in question is the causative variant. In light of the mild KdVS phenotype reported in individual EX18, future studies investigating genotype-phenotypes correlations in individuals with missense *KANSL1* variants will be critical.

The architecture of the 17q21.31 locus poses additional diagnostic challenges due to the genomic complexity and structural diversity of 17q21.31 region. There are two haplotypes reported in the human population, H1 and H2, associated with a longer and shorter segmental duplications, respectively, that overlap the promoter and first three exons of *KANSL1* [9]. Both haplotypes are present in European and Mediterranean populations at frequencies as high as 60% [10, 39]. During genetic testing, the genomic complexity of the 17q21.31 region can result in ‘artefacts’, which complicates the mapping and interpretation of both copy number and single nucleotide variants. Inherited and/or benign 17q21.31 CNVs as well as loss-of-function (LoF) single nucleotide variants within exons 1–3 of *KANSL1* are reported in databases such as gnomAD (https://gnomad.broadinstitute.org/). One example is the variant in exon 2 identified in two individuals EX10 and EX11 [p.(Leu270Valfster11)] and is reported in gnomAD seven times. In such cases it is important to assess the phenotype carefully. Both individuals EX10 and EX11 were included in the signature validation cohort and were reported to have a phenotype in-line with KdVS. These phenotypic findings correlated with a pathogenicity score >77% for both individuals EX10 and EX11, confirming their DNAm profile as ‘KdVS-like’. Another example is the nonsense variant p. (Arg290ter) reported in gnomAD 16 times and found in individual EX16 included in the validation cohort. The clinical presentation of individual EX16 was also in-line with KdVS and was further supported by a KdVS-like DNAm classification score (76%) confirming the KdVS diagnosis. It is possible that these LoF variants in the first 3 exons of *KANSL1* reported in control populations are present in duplicated, non-functional copies of *KANSL1* with no clinical consequences [40]. Currently careful clinical investigation in combination with DNA methylation testing is the only method that can discriminate whether a variant is KdVS-causing in this region.

Studies have shown a role for the NSL complex in regulating H4K16 acetylation of autophagy related genes [41]. Autophagy is shown to have a critical physiological role in neuronal health and function [42]. In KANSL1-deficient human induced pluripotent stem cells from KdVS patients, loss of KANSL1 resulted in autophagosome accumulation due to increased oxidative stress [43]. One of the top differentially methylated genes in the KdVS signature is *ORMDL3*, or sphingolipid biosynthesis regulator 3, found to promote autophagy in epithelial cells [44]. Other genes implicated in autophagy in the KdVS signature include *ATG2A* and *EPAS1* [45]. The KdVS signature also includes many CpGs within key genes that regulate neuronal function and development: including but not limited to *SHANK2* and *NEURL*. Differentially methylated genes in the KdVS DNAm signature are reflective of syndrome pathophysiology in that many of the genes are implicated in KANSL1 related functions and are likely affected by *KANSL1* haploinsufficiency.

While our study provides demonstrates the utility of the KdVS DNAm signature in variant classification and diagnosis, there are some limitations that should be acknowledged. First, our study focused on peripheral blood-derived DNAm profiles, which may not fully capture tissue-specific epigenetic changes that could be relevant to KdVS pathophysiology. Second, the functional relevance of the signature genes in the context of *KANSL1*-related functions and KdVS pathophysiology was explored through in silico predictions and protein modeling. Further experimental studies, such as functional assays and gene expression analyses, are required to validate the functional impact of these genes and their potential contribution to KdVS phenotypes.

In conclusion, we report a unique DNAm signature that is highly sensitive and specific for *KANSL1* haploinsufficiency and KdVS. The generated KdVS DNAm signature can be used as an additional molecular test to address the diagnostic challenges associated with the 17q21.31 locus. For future studies, additional functional studies of missense variants in *KANSL1* paired with DNAm profiling will be important for furthering our understanding of molecular mechanisms underpinning this syndrome. In addition, in vitro models of induced pluripotent stem cells derived combined with multi-omics approaches will further elucidate molecular and epigenetic changes associated with *KANSL1* variants causing KdVS.

### Supplementary information

Supplementary Fig. 1

Supplementary Fig. 2

Supplementary Fig. 3

Supplementary Fig. 4

Supplementary Tables

Supplementary Figures Caption

## Data Availability

The datasets generated during the current study are not publicly available due to institutional ethical restrictions but are available from the corresponding author on reasonable request.
