# Correction: A new blood DNA methylation signature for Koolen-de Vries syndrome: Classification of missense *KANSL1* variants and comparison to fibroblast cells

**DOI:** 10.1038/s41431-024-01561-7

**Published:** 2024-02-15

**Authors:** Zain Awamleh, Sanaa Choufani, Wendy Wu, Dmitrijs Rots, Alexander J. M. Dingemans, Nael Nadif Kasri, Susana Boronat, Salvador Ibañez-Mico, Laura Cuesta Herraiz, Irene Ferrer, Antonio Martínez Carrascal, Luis A. Pérez-Jurado, Gemma Aznar Lain, Juan Dario Ortigoza-Escobar, Bert B. A. de Vries, David A. Koolen, Rosanna Weksberg

**Affiliations:** 1 Genetics and Genome Biology Program, Research Institute, the Hospital for Sick Children, Toronto, ON M5G 1×8 Canada; 2 Department of Human Genetics, Radboud University Medical Center, Donders Institute for Brain, Cognition, and Behavior, Nijmegen, The Netherlands; 3 Department of Pediatrics, Hospital del Santa Creu y Sant Pau, Barcelona, Spain; 4 Department of Pediatric Neurology, Hospital Virgen de la Arrixaca, Murcia, Madrid Spain; 5 Department of Pediatric Neurology, Hospital de Manises, Valencia, Spain; 6 Department of Genetics, Consorcio Hospital General de Valencia, Valencia, Spain; 7 Department of Pediatrics, Hospital de Requena, Valencia, Spain; 8 Genetics Unit, Universitat Pompeu Fabra, Hospital del Mar Research Institute (IMIM) and CIBERER, Barcelona, Spain; 9 Movement Disorders Unit, Institut de Recerca Sant Joan de Déu, CIBERER-ISCIII and European Reference Network for Rare Neurological Diseases (ERN-RND), Barcelona, Spain; 10 Division of Clinical and Metabolic Genetics, Department of Pediatrics, the Hospital for Sick Children, University of Toronto, Toronto, ON M5G 1×8 Canada

**Keywords:** DNA methylation, Diagnostic markers

Correction to: *European Journal of Human Genetics* 10.1038/s41431-024-01538-6, published online 29 January 2024.

In this article, the author name Nael Nadif Kasri was incorrectly written as Nael Nadif Khadri.

The original article has been corrected.

