# Supplementary figures and images for: A new blood DNA methylation signature for Koolen-de Vries syndrome: Classification of missense KANSL1 variants and comparison to fibroblast cells
Source: Eur J Hum Genet. 2024 Jan 29;32(3):324–32. doi: 10.1038/s41431-024-01538-6 (PMC10923882; doi:10.1038/s41431-024-01538-6)

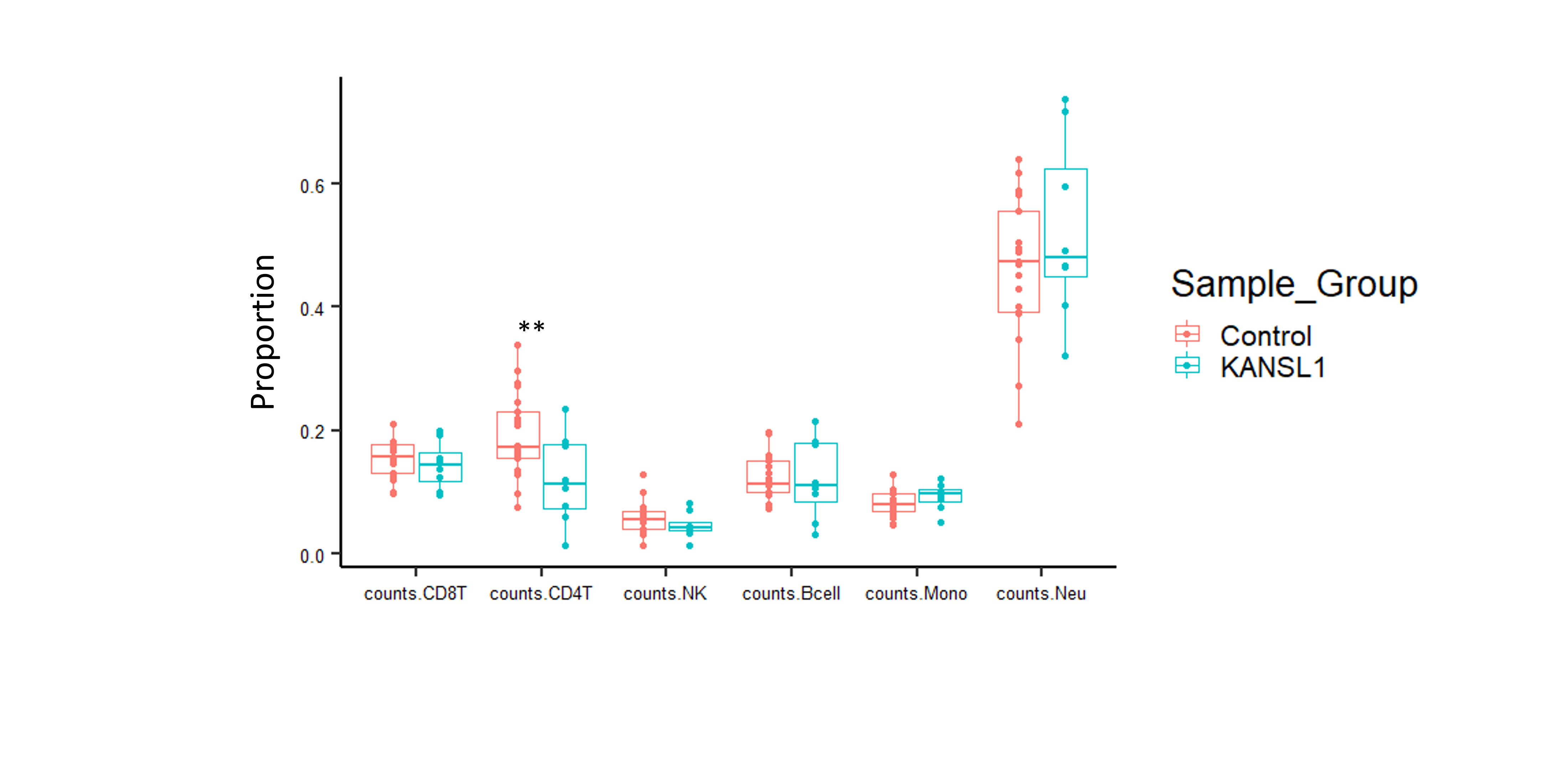

Supplement: Supplementary file 1 — Supplementary Fig. 1 [file 41431_2024_1538_MOESM1_ESM.png]

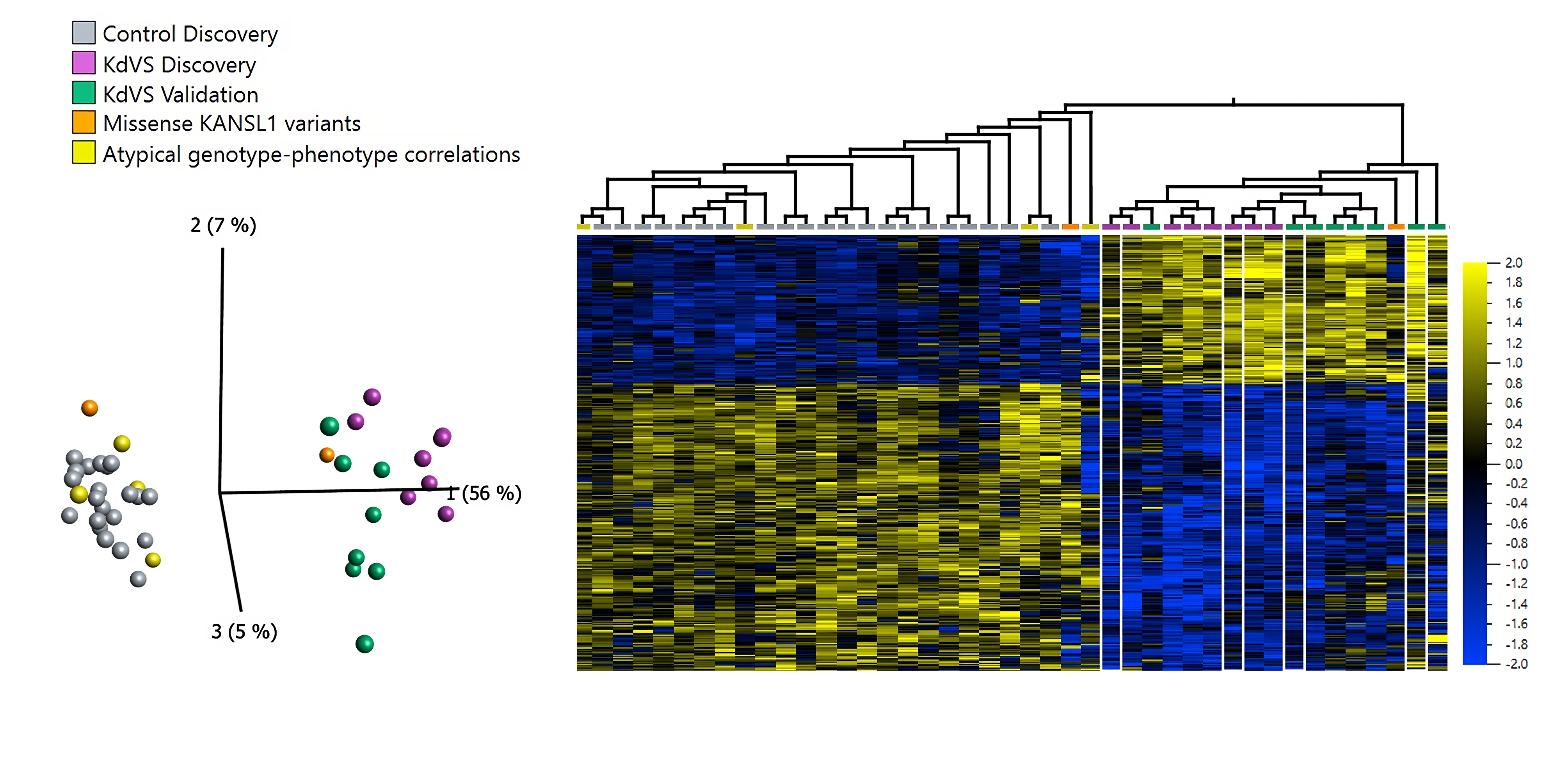

Supplement: Supplementary file 2 — Supplementary Fig. 2 [file 41431_2024_1538_MOESM2_ESM.png]

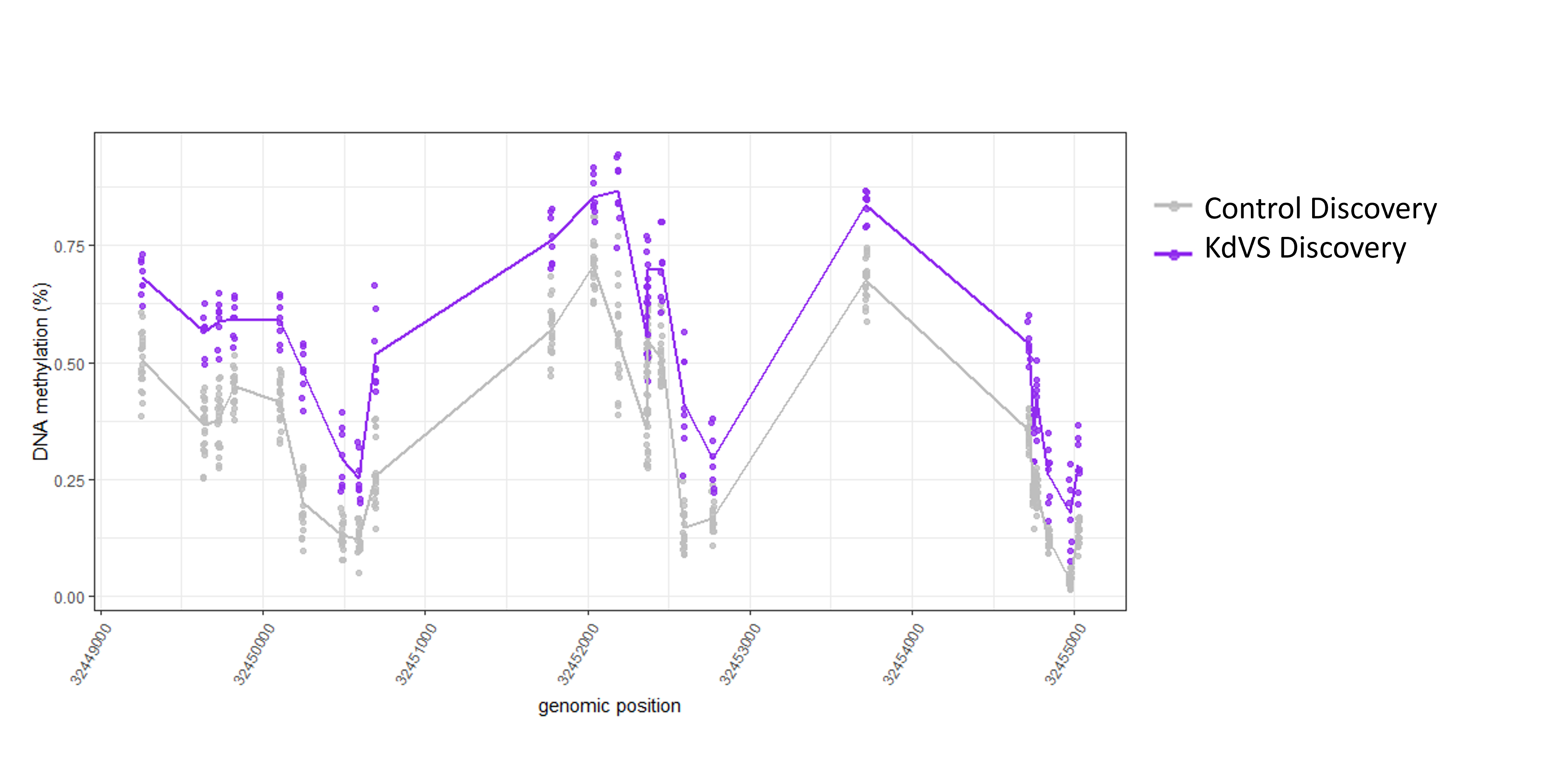

Supplement: Supplementary file 3 — Supplementary Fig. 3 [file 41431_2024_1538_MOESM3_ESM.png]

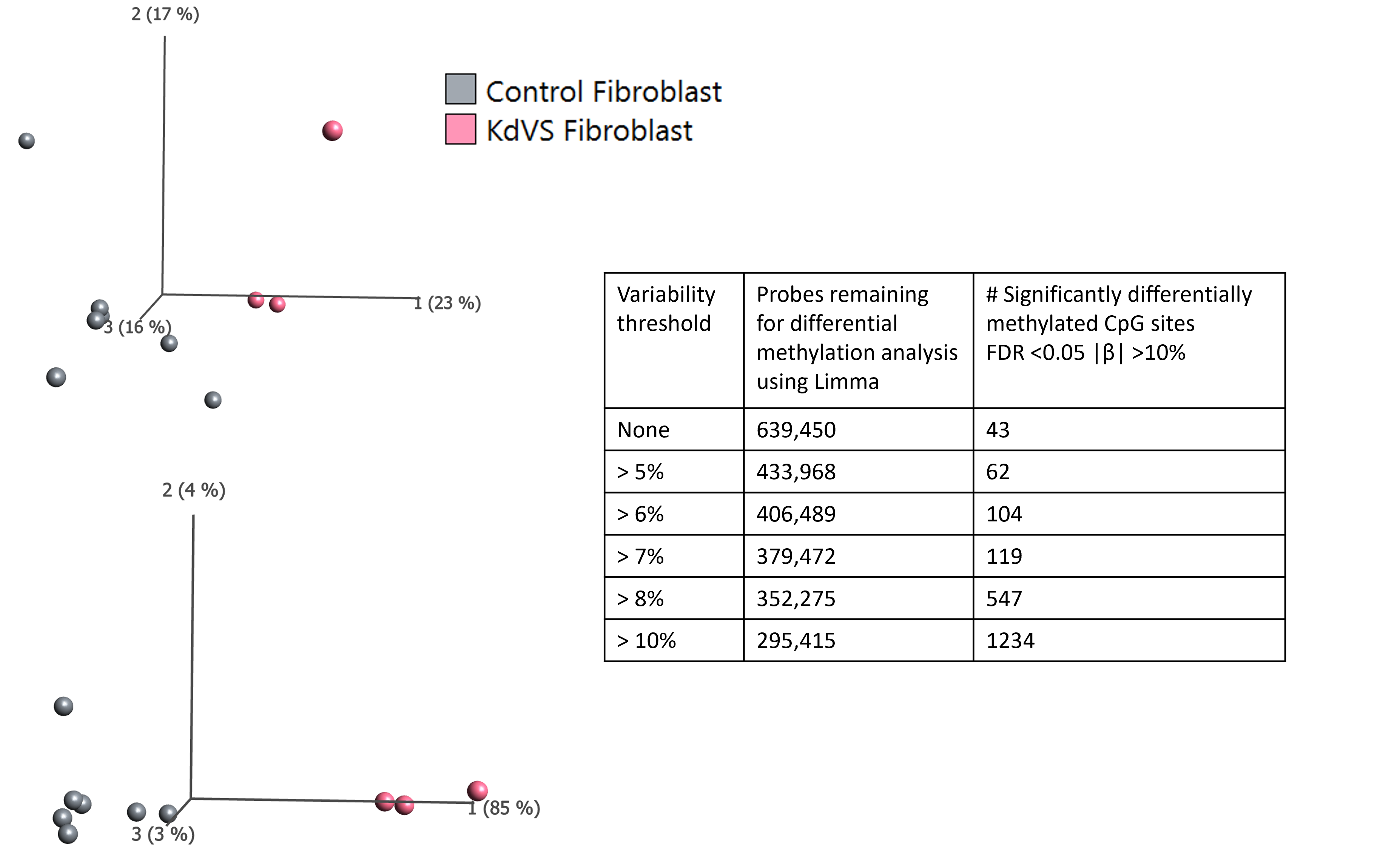

Supplement: Supplementary file 4 — Supplementary Fig. 4 [file 41431_2024_1538_MOESM4_ESM.png]
