# Supplementary material for: A new blood DNA methylation signature for Koolen-de Vries syndrome: Classification of missense KANSL1 variants and comparison to fibroblast cells
Source: Eur J Hum Genet. 2024 Jan 29;32(3):324–32. doi: 10.1038/s41431-024-01538-6 (PMC10923882; doi:10.1038/s41431-024-01538-6)
Supplement: Supplementary file 6 — Supplementary Figures Caption [file 41431_2024_1538_MOESM6_ESM.docx]

**Supplementary Figure 1. Estimated cell type proportions.** We applied the blood cell-type proportion estimation tool in minfi based on Illumina EPIC array data from FACS-sorted blood cells. Mean cell type proportion is shown for each cell type with each individual represented with a closed dot. Controls are indicated in red and KdVS cases are indicated in blue. Statistical significance assessed using an unpaired two-tail t-test (**p<0.01)

**Supplementary Figure 2. Validation of KdVS DNAm signature in an independent cohort.** (A) Principal component analysis (PCA) and (B) heatmap showing clustering of KdVS discovery (n=8; purple) and validation (n=7; green) subjects, along with controls (n=21; grey) and subjects considered for classification (n=6; orange/yellow) using DNAm values at the 456 CpG sites identified in the KdVS DNAm signature. The four profiles with white rectangles belong to the four individuals with microdeletions at 17q21.31 which were split between discovery and validation cohorts. The heatmap color gradient indicates the normalized DNAm value ranging from -2.0 (blue) to 2.0 (yellow). Euclidean distance metric is used in the heatmap clustering dendrograms.

**Supplementary Figure 3. Signature CpG sites overlapping the WT1 gene.** Graph represents the genomic location of the CpG site on the x-axis and the percentage of methylation difference on the y-axis between controls (grey) and KdVS cases (purple). There are 24 CpG sites in the KdVS signature, all of which are hypermethylated (12% to 31%) and represented in the figure.

**Supplementary Figure 4. Further analysis of DNA methylation changes in fibroblast cells.** (A) PCA plot of fibroblast samples clustered based on the blood KdVS DNAm signature (456 CpG sites). (B) Table of remaining probes after filtering non-variable probes at different variability thresholds and the number of significant CpG sites identified. (C) PCA plot of fibroblast samples clustered based on 1234 significant CpG sites identified after removing non-variable probes at a 10% threshold.
